# Engineering the hydroxyl content on aluminum oxyhydroxide nanorod for elucidating the antigen adsorption behavior

**DOI:** 10.1038/s41541-022-00495-9

**Published:** 2022-06-23

**Authors:** Ge Yu, Zhihui Liang, Zilan Yu, Min Li, Wenqi Yang, Yawei Zhang, Yuhang Zhao, Cheng Yang, Changying Xue, Li Shi, Bingbing Sun

**Affiliations:** 1 State Key Laboratory of Fine Chemicals, Dalian University of Technology, 2 Linggong Road, 116024 Dalian, China; 2 School of Chemical Engineering, Dalian University of Technology, 2 Linggong Road, 116024 Dalian, China; 3 School of Chemistry, Dalian University of Technology, 2 Linggong Road, 116024 Dalian, China; 4 School of Bioengineering, Dalian University of Technology, 2 Linggong Road, 116024 Dalian, China; 5 Immune Path Biotechnology (Su Zhou) Co., Ltd., Building A, 8 Chang Ting Road, DaXin Industry Park, 215151 Su Zhou, Jiang Su China

**Keywords:** Adjuvants, Biotechnology

## Abstract

The interaction between the aluminum salt-based adjuvants and the antigen in the vaccine formulation is one of the determining factors affecting the immuno-potentiation effect of vaccines. However, it is not clear how the intrinsic properties of the adjuvants could affect this interaction, which limits to benefit the improvement of existing adjuvants and further formulation of new vaccines. Here, we engineered aluminum oxyhydroxide (AlOOH) nanorods and used a variety of antigens including hepatitis B surface antigen (HBsAg), SARS-CoV-2 spike protein receptor-binding domain (RBD), bovine serum albumin (BSA) and ovalbumin (OVA) to identify the key physicochemical properties of adjuvant that determine the antigen adsorption at the nano-bio interface between selected antigen and AlOOH nanorod adjuvant. By using various physicochemical and biophysical characterization methods, it was demonstrated that the surface hydroxyl contents of AlOOH nanorods affected the adsorptive strength of the antigen and their specific surface area determined the adsorptive capacity of the antigen. In addition, surface hydroxyl contents had an impact on the stability of the adsorbed antigen. By engineering the key intrinsic characteristics of aluminum-based adjuvants, the antigen adsorption behavior with the aluminum adjuvant could be regulated. This will facilitate the design of vaccine formulations to optimize the adsorption and stability of the antigen in vaccine.

## Introduction

Aluminum-based adjuvant (Alum) is a critical component in existing subunit, toxoid, and certain inactivated vaccines. It can improve the immunogenicity of vaccines and enhance the antigen-specific immune responses^1–3^. When Alum is formulated with antigens, the interactions between the adjuvant and the antigen determine the degree of adsorption and affect the stability of the antigen, which further affect vaccine’s immunogenicity^4–7^. Therefore, controlling antigen adsorption on adjuvants is an effective strategy to optimize immunological effects of vaccines.

Studies have been focused on the modifications of antigens to enhance the affinity with Alum, and the common method is the phosphorylation of antigens by the chemical modification of proteins with phosphoserine (pSer)^8^ or phosphonate groups (C-PO_3_)^9^. However, the modification of antigen involves delicate processes and might affect the inherent structure and immunogenicity of the antigen. Alternatively, modification of the physicochemical properties of Alum were shown to promote optimized antigen adsorption and further immunological effects. For instance, Hem et al. showed that the adsorptive strength of phosphate-treated adjuvants to hepatitis B surface antigen (HBsAg) and HIV 1 gp140 antigens would be reduced, thereby triggering a stronger immune response^5^^,10^. Egan et al. combined aluminum hydroxyl phosphate sulfate with fluoride or phosphate to induce antigen desorption, and it was shown that the reduction of binding strength was beneficial to enhance the immunogenicity^11^. However, the above two cases are both testing to reduce the adsorptive strength of the Alum adjuvant for the selected antigens, the intrinsic physicochemical properties that determine antigen adsorption are not clear, and there is no systematic control or related information available for the commercially available Alum, which leaves less room for the optimization and development of new vaccine formulations.

Engineered nanomaterials exhibit well-controlled characteristics, and can be used as vaccine adjuvants to achieve optimized immunogenicity^12–15^. Thus, it is a good strategy to engineer the physicochemical properties of nanomaterials to regulate antigen adsorption behavior. For example, Clemments et al. fabricated silica nanoparticles with different size, and further incubated them in FBS. It was found that smaller particles adsorbed a larger amount of protein due to the larger external surface area^16^. Feng et al. studied the adsorption of bovine serum albumin (BSA) on the surface of titanium with different oxide films and showed positive correlation of BSA adsorption with hydroxyl group contents on the titanium surface^17^. While compared with amorphous titanium dioxide (TiO_2_) nanodots, Hong et al. demonstrated that, BSA exhibited multilayer adsorption on anatase TiO_2_ nanodots with fewer surface hydroxyl groups^18^. These contradictory results indicate the complexity of antigen adsorption on different nanoparticles, making it difficult to understand the structure-activity relationship between physicochemical properties of adjuvants and antigen adsorption mechanistically.

In addition, not only the degree of adsorption between the adjuvant and the antigen, but also the possible conformation changes of the antigen on the Alum will affect the immune effects. Thalhamer et al. demonstrated that the loss of antigen conformational stability could lead to reduced antibody production^19^. D’Souza et al. showed that the deamidation of recombinant anthrax vaccine antigen rPA adsorbed on aluminum hydroxide was the direct cause of the reduced vaccine efficacy^20^. Therefore, a mechanism study is necessary to have a better understanding of Alum property-related changes in the structure and stability of antigen.

In this study, by controlling the surface hydroxyl contents and specific surface area of AlOOH nanorods, the adsorption isotherms of a variety of antigens, e.g., HBsAg, SARS-CoV-2 spike protein receptor-binding domain (RBD), BSA, and ovalbumin (OVA) with engineered nanoadjuvants were established to explore the interaction mechanisms. After the adsorption of the antigen to the AlOOH nanorods, changes in the structure and thermal stability of the antigen were further evaluated. The correlation between the key physicochemical properties of adjuvants and the adsorption behavior has been elucidated for the first time. It provides a foundation for the engineered design of nanomaterial-based adjuvants for both prophylactic and therapeutic vaccine formulations.

## Results and discussion

### Characterization of AlOOH nanorods

**Fig. 1:**
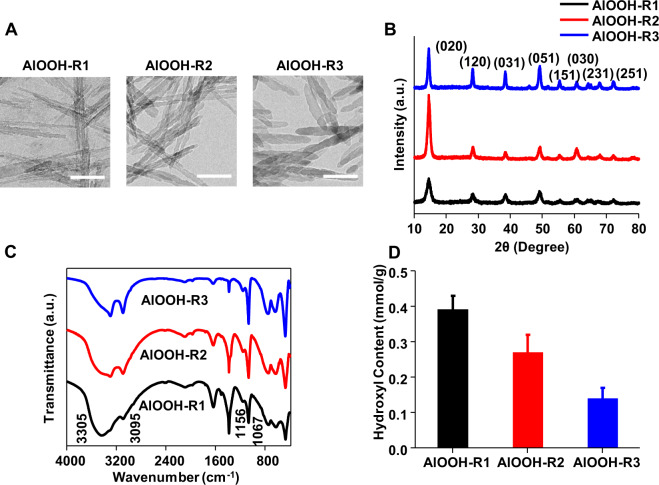
Characterization of engineered AlOOH nanorods. **A** TEM images of AlOOH nanorods synthesized at a temperature of 160 °C (R1), 180 °C (R2), and 200 °C (R3). The scale bar is 100 nm. **B** XRD analysis of engineered AlOOH nanorods. **C** FTIR spectra of AlOOH nanorods. **D** The surface hydroxyl contents of AlOOH nanorods.

A library of engineered AlOOH nanorods with controlled physicochemical properties was prepared by using hydrothermal method. Transmission electron microscopy (TEM) showed that the AlOOH nanorods exhibited a uniform rod-like morphology with a dimension of 219 ± 36 nm in length and 10 ± 2 nm in diameter (Fig. 1A, Supplementary Table 1). The hydrodynamic sizes of AlOOH nanorods were in between 200–300 nm in water, and the zeta potential measurement in water demonstrated that AlOOH nanorods exhibited positive charges at 42 ± 2 mV, 49 ± 2 mV, and 50 ± 3 mV for R1, R2, and R3, respectively (Table 1). In comparison, Alhydrogel^®^ exhibited similar rod-like morphology and showed hydrodynamic size of 503 ± 9 nm and zeta potential of 27 ± 2 mV in water (Supplementary Figure 1 and Table 2). XRD analysis showed that the AlOOH nanorods were boehmite and did not show any impurity diffraction peaks (Fig. 1B). FTIR spectra of AlOOH nanorods exhibited characteristic bands of AlOOH (Fig. 1C). The two bands at 3300 and 3095 cm^−1^ were assigned to the asymmetric (ν_as_(Al)O-H) and symmetric (ν_s_(Al)O-H) stretching vibrations of the OH group. The two bands at 1156 and 1067 cm^−1^ were attributed to asymmetric (ν_as_Al-O-H) and symmetric (ν_s_Al-O-H) OH deformation^12^. As the synthesis temperature increased, the width at half height (WHH) of the (020) reflection gradually decreased from 1.33 to 0.66 (Table 1), indicating the increase of crystallinity. Potentiometric titration of hydroxyl contents showed that the hydroxyl contents on the surface of AlOOH nanorods decreased from 0.39 mmol/g to 0.14 mmol/g from R1 to R3 (Table 1, Fig. 1D and Supplementary Figure 2). The reduction of surface hydroxyl content was further confirmed by the zeta potential measurement at neutral pH, which were 37 ± 1 mV, 32 ± 2 mV, and 25 ± 3 mV for R1, R2, and R3, respectively (Supplementary Table 3). Similarly, the surface area decreased from 164.8 m^2^/g to 85.1 m^2^/g. As a control, Alhydrogel^®^ also exhibited a higher specific surface area of 270.8 m^2^/g (Supplementary Table 2), however, the content of surface hydroxyl could not be quantitatively determined by potentiometric titration. The points of zero charge (PZC) measurement demonstrated that the engineered AlOOH nanorods exhibited PZCs of 9.46–9.72 under physiological conditions (Table 1). They were similar to that of Alhydrogel^®^, whose PZC was reported to be 9.61 (Supplementary Table 2).

**Table 1:** Characterizations of AlOOH nanorods.

Sample ID	Hydrodynamic size in water (nm)	Zeta potential in water (mV)	PZC	Specific surface area (m^2^/g)	Hydroxyl content (mmol/g)	WHH (2θ)
AlOOH-R1	270 ± 2	42 ± 2	9.72 ± 0.00	164.8 ± 3.8	0.39 ± 0.04	1.33 ± 0.04
AlOOH-R2	263 ± 2	49 ± 2	9.51 ± 0.01	122.2 ± 1.0	0.27 ± 0.05	0.83 ± 0.02
AlOOH-R3	220 ± 1	50 ± 3	9.46 ± 0.00	85.1 ± 5.8	0.14 ± 0.03	0.66 ± 0.01

**Table 2:** Adsorption parameters for HBsAg, RBD, OVA, and BSA by AlOOH nanorods.

Antigen	Adjuvant	Adsorptive capacity (mg/mgAl)	Coefficient of determination (R^2^)	Adsorptive coefficient (mL/mg)	Monolayer adsorptive capacity (mg/mgAl)
HBsAg	AlOOH-R1	2.42 ± 0.03	0.99 ± 0.00	1319 ± 324	2.38 ± 0.06
	AlOOH-R2	1.74 ± 0.14	0.99 ± 0.00	219 ± 24	1.76 ± 0.15
	AlOOH-R3	1.35 ± 0.08	0.97 ± 0.01	59 ± 1	1.44 ± 0.02
BSA	AlOOH-R1	1.12 ± 0.07	0.99 ± 0.00	37 ± 2	1.10 ± 0.07
	AlOOH-R2	0.56 ± 0.11	0.99 ± 0.01	24 ± 8	0.55 ± 0.14
	AlOOH-R3	0.38 ± 0.00	0.96 ± 0.05	3 ± 1	0.40 ± 0.00
OVA	AlOOH-R1	1.04 ± 0.06	0.99 ± 0.00	21 ± 5	1.05 ± 0.06
	AlOOH-R2	0.82 ± 0.02	0.99 ± 0.01	17 ± 1	0.82 ± 0.05
	AlOOH-R3	0.59 ± 0.01	0.98 ± 0.01	4 ± 1	0.58 ± 0.05
RBD	AlOOH-R1	0.62 ± 0.02	0.99 ± 0.00	147 ± 19	0.65 ± 0.01
	AlOOH-R2	0.55 ± 0.01	0.99 ± 0.00	87 ± 20	0.58 ± 0.01
	AlOOH-R3	0.44 ± 0.01	0.97 ± 0.01	56 ± 7	0.49 ± 0.01

### Adsorption isotherms of antigens by AlOOH nanorods

The engineered AlOOH nanorods showed a positive charge at neutral pH, thus HBsAg (pI = 6.85), RBD (pI = 7.68), BSA (pI = 4.7~5.3), and OVA (pI = 4.4–4.9), were selected as model antigens for adsorption studies. The 3-(N-morpholino) propanesulfonic acid (MOPS) was selected as adsorption buffer, and the Langmuir equation was used to describe adsorption isotherms. The linear fits showed a minimum value R^2^ of 0.96, which seemed to indicate that the Langmuir equation could describe the adsorption of the model antigens by the AlOOH nanoadjuvants (Table 2). But it is worth noting that there are four assumptions to describe the adsorption process with the Langmuir adsorption isotherm: (i) all adsorption sites are equivalent and independent, (ii) each adsorption site can only bind one solute molecule, (iii) there is no interaction between adsorbed solute molecules, (iv) the adsorption process must be dynamically reversible^21,22^. Due to the complexity of protein adsorption to the solid surface, although the Langmuir equation fitted the protein adsorption curve well, the protein adsorption process usually deviated greatly from the assumption of Langmuir adsorption behavior, and the obtained fitting parameters may produce errors. Therefore, the adsorption parameters between adjuvant and antigen were verified by isothermal titration calorimetry (ITC) that will be discussed in later section.

**Fig. 2:**
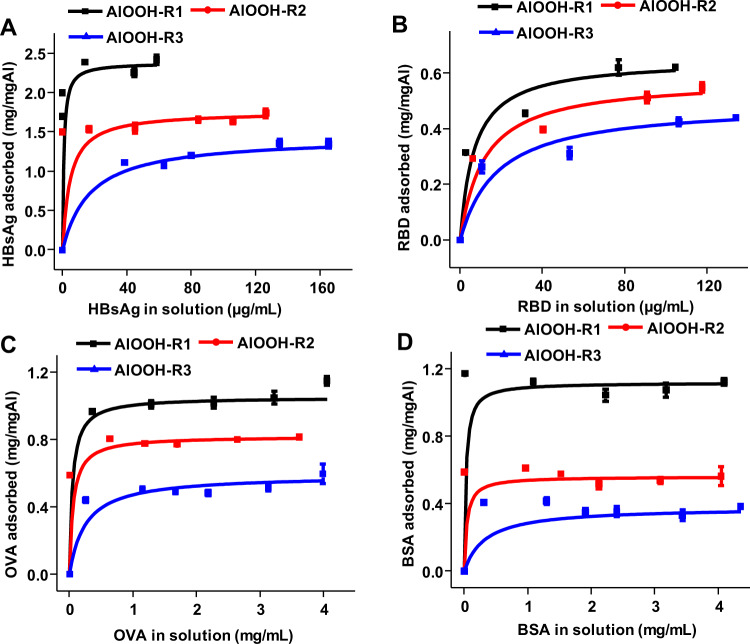
Adsorption isotherms of antigens by AlOOH. The adsorption isotherms of **A** HBsAg, **B** RBD, **C** OVA, and **D** BSA by AlOOH nanorods in MOPS buffer at pH 7.4.

In 10 mM of MOPS buffer (pH 7.4)^5^, the adsorptive capacity of AlOOH nanorods to model antigens showed that the amount of adsorbed antigen gradually increased with the increase of antigen concentration until it reached a steady state. As the surface area and surface hydroxyl contents decreased from R1 to R3, the adsorptive capacity decreased from 1.12 to 0.38 mg/mg Al for BSA, from 1.04 to 0.59 mg/mg Al for OVA, from 2.42 to 1.35 mg/mg Al for HBsAg, and from 0.62 to 0.44 mg/mg Al for RBD (Fig. 2A–D, Table 2). The calculated adsorptive coefficient also decreased from 37 mL/mg, 21 mL/mg, 1319 mL/mg and 147 mL/mg to 3 mL/mg, 4 mL/mg, 59 mL/mg and 56 mL/mg for the corresponding model antigens (Fig. 2A–D, Table 2). It can be seen that the adsorptive capacities and adsorptive coefficients of all antigens decreased significantly from R1 to R3 (Supplementary Table 4). Similarly, the adsorption of four model antigens on Alhydrogel^®^ could also be fitted by Langmuir isotherm equation (Supplementary Figure 3, Supplementary Table 5). It is notable that although different doses of adjuvant and antigen would affect the adsorption behavior, it did not affect the trend of adsorptive coefficients and adsorptive capacities (Supplementary Table 6).

### AlOOH nanorod physicochemical property-dependent antigen adsorption

**Fig. 3:**
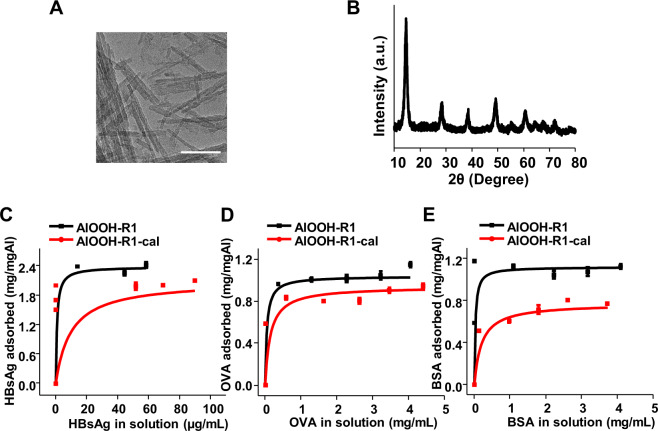
Effect of specific surface area and surface hydroxyl content of AlOOH nanorods on antigen adsorption. **A** TEM analysis and **B** XRD patterns of the AlOOH-R1 after calcination. AlOOH-R1-cal was obtained by treating AlOOH nanorods at 350 °C for 1 h. The scale bar is 100 nm. Adsorption isotherms of **C** HBsAg, **D** OVA, and **E** BSA by AlOOH-R1 and AlOOH-R1-cal in MOPS buffer at pH 7.4.

Engineered AlOOH nanorods exhibited similar physicochemical properties, including size, charge, but different specific area and surface hydroxyl contents (Table 1, Supplementary Figure 1). In order to determine the effects of surface area and surface hydroxyl content on antigen adsorption, R1 with the highest hydroxyl content was placed in a muffle furnace at 350 °C to proportionally remove surface hydroxyl content while maintaining its surface area. After calcination, it remained as boehmite^12^, and there was no significant change in zeta potential, the hydrodynamic size increased to around 400 nm in water (Fig. 3A, B and Supplementary Figure 4). The specific surface area dropped from 164.8 m^2^/g to 149.2 m^2^/g, a reduction of 9.5%. In comparison, the surface hydroxyl contents decreased from 0.39 mmol/g to 0.08 mmol/g, a reduction of 79%. Additionally, the zeta potential measurement at neutral pH indicated that the calcination processed reduced the zeta potential, which further contributed to the increase of hydrodynamic sizes after calcination (Supplementary Table 3). According to the statistical analysis, with the substantial decrease in the amount of surface hydroxyl contents, the adsorptive strength of HBsAg, BSA and OVA by AlOOH nanorods decreased significantly (Fig. 3C–E and Table 3 and Supplementary Table 7). In comparison, the adsorptive capacities remained or changed in a lesser degree (Fig. 3C–E and Table 3 and Supplementary Table 7). These results suggested that the surface hydroxyl groups on AlOOH nanorods could affect the adsorptive strength, while the specific surface area of particles could be correlated to the adsorptive capacity.

**Table 3:** Effect of specific surface area and surface hydroxyl contents on the adsorptive capacity and adsorptive coefficient of HBsAg, BSA, and OVA by R1 and calcinated R1 nanorods.

Antigen	Adjuvant	Hydroxyl content (mmol/g)	Specific surface area (m^2^/g)	Adsorptive capacity (mg/mgAl)	Adsorptive coefficient (mL/mg)
HBsAg	AlOOH-R1	0.39 ± 0.04	164.8 ± 3.8	2.42 ± 0.03	1319 ± 324
	AlOOH-R1-cal	0.08 ± 0.01	149.2 ± 7.8	2.10 ± 0.14	101 ± 6
BSA	AlOOH-R1	0.39 ± 0.04	164.8 ± 3.8	1.12 ± 0.07	37 ± 2
	AlOOH-R1-cal	0.08 ± 0.01	149.2 ± 7.8	0.77 ± 0.03	5 ± 2
OVA	AlOOH-R1	0.39 ± 0.04	164.8 ± 3.8	1.04 ± 0.06	21 ± 5
	AlOOH-R1-cal	0.08 ± 0.01	149.2 ± 7.8	0.94 ± 0.02	7 ± 4

**Fig. 4:**
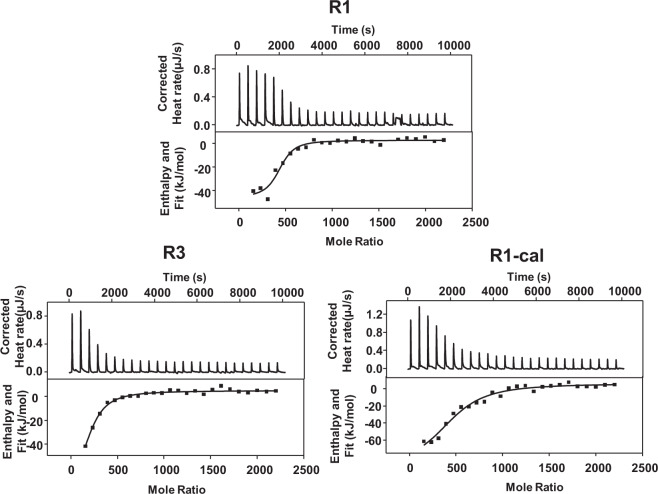
ITC analysis of the titration of OVA to AlOOH nanorods. Heat flow versus time during the injection of OVA over AlOOH nanorods (top panel) and heat evolved per mole of added OVA against the molar ratio per injection (bottom panel) for **A** R1, **B** R3, and **C** R1-Cal. All titrations were performed at a pH of 7.4 and 25 °C. Calcinated nanorods R1 were prepared at 350 °C in a muffle furnace for one hour.

In order to further confirm the interaction between AlOOH nanorods and model antigens, ITC was used to characterize the adsorption behavior of antigen on AlOOH nanorods (Fig. 4).

OVA was selected as a model antigen. R1, R3, and calcinated R1 were chosen to study the adsorption behavior. With the decrease of surface hydroxyl, the dissociation constant (K_d_) was increased. The number of adsorbed OVA per nanoparticle (N) was decreased for R3, but remained unchanged for calcinated R1. It was consistent with the fitting results of the Langmuir adsorption model (Table 5, Table 4), suggesting that it was the surface hydroxyl rather than the specific surface area that affected the adsorptive coefficient of the antigen to the AlOOH nanorods. It was worth noting that although the binding constant (K_I_) measured by ITC and calculated by Langmuir equation (K_ads_) were very similar, the stoichiometry obtained by ITC (N_I_) and Langmuir equation (N_ads_) were quite different (Table 5). It suggested that a part of the adsorbed OVA did not produce heat changes, and some binding sites did not show measurable enthalpy changes^23^. Another possible reason was that OVA and AlOOH nanorods formed aggregates after mixing (Supplementary Figure 5)^23^, which prevented the release of unbound and loosely bound proteins. Thus, the actual amount of adsorbed protein was overestimated, and further caused a deviation in the fitting of the Langmuir equation.

**Table 4:** Thermodynamic parameters measured by ITC for the binding of OVA to R1, R3, and calcinated R1 nanorods at 25 °C.

Sample ID	K_d_ (10^−7^ M)	*n*	ΔH (kJ/mol)	ΔS (J/mol K)	ΔG (kJ/mol)
AlOOH-R1 + OVA	7.63 ± 7.42	414 ± 35	−49 ± 8	−47 ± 19	−35 ± 1
AlOOH-R3 + OVA	32.8 ± 9.47	176 ± 27	−97 ± 17	−220 ± 7	−31 ± 0
AlOOH-R1-cal+OVA	54.0 ± 1.82	466 ± 47	−98 ± 11	−228 ± 32	−30 ± 1

**Table 5:** Adsorption constants and amounts of adsorbed protein per NP measured by ITC (K_I_ and N_I_) and Langmuir adsorption isotherms (K_ads_ and N_ads_).

Sample ID	K_I_ (10^5^ M^−1^)	K_ads_ (10^5^ M^−1^)	N_I_ (protein per NP)	N_ads_ (protein per NP)
AlOOH-R1 + OVA	13.1	9.25	414	770
AlOOH-R3 + OVA	3.04	1.87	176	437
AlOOH-R1-cal+OVA	1.85	3.29	466	696

**Fig. 5:**
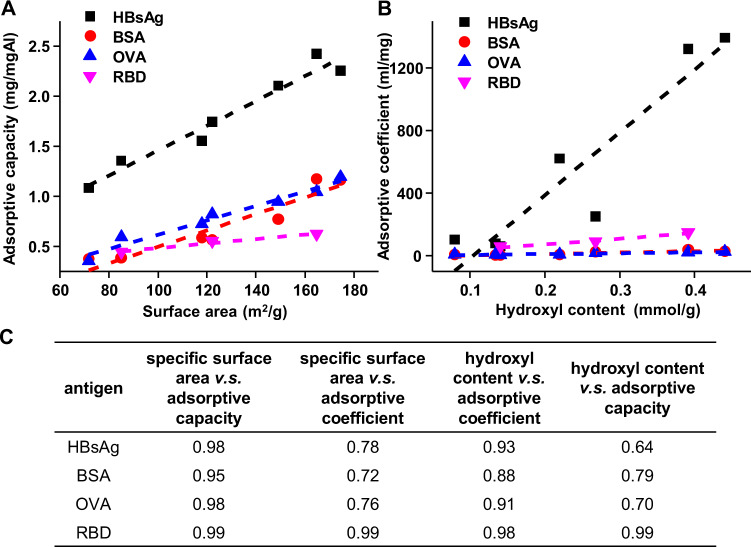
Correlation between physicochemical properties of AlOOH nanorods and antigen adsorption. **A** The relationship between the specific surface area of AlOOH nanorods and adsorptive capacity. **B** The relationship between the surface hydroxyl contents of AlOOH nanorods and adsorptive coefficient. **C** The Pearson correlation coefficient between the physicochemical properties of AlOOH nanorods and the antigen adsorption parameters.

Additionally, another library of rod-shaped AlOOH nanoparticles was used to validate the role of hydroxyl content in antigen adsorption (Supplementary Figure 6). The surface hydroxyl content was tuned by controlling the synthesis time that had been reported in our previous study (Supplementary Table 8)^12^. The effect of hydroxyl content and surface area on antigen adsorption were examined by using three model antigens, i.e., BSA, OVA, and HBsAg. The adsorptive capacities and adsorptive coefficients of BSA, OVA, and HBsAg decreased with the decrease of the surface hydroxyl contents and surface area (Supplementary Figure 7, Supplementary Table 9)^24^. Furthermore, the amount of surface hydroxyl contents on nanorods from these two engineered nanoadjuvant libraries was correlated with the adsorptive coefficients of BSA, OVA, HBsAg, and RBD, while the surface area was correlated with the adsorptive capacities of antigens (Fig. 5). It was demonstrated that there exhibited a good correlation between surface hydroxyl content and antigen adsorptive strength. In comparison, the surface area showed better correlation with antigen adsorptive capacity (Fig. 5).

It’s interesting to note that the adsorptive coefficients of BSA, OVA, and RBD were very different from that of HBsAg. For BSA, OVA, and RBD, the adsorptive coefficients were in the order of tens. In comparison, the adsorptive coefficient of HBsAg was in the order of thousands (Table 2), suggesting the adsorption mechanisms of HBsAg on AlOOH nanorods was different from that of BSA, OVA, and RBD. It has been demonstrated that the interaction between HBsAg and aluminum-based adjuvants was mainly through ligand exchange^5,25^, while for OVA, BSA, and RBD, they could bind to AlOOH nanorods through electrostatic interaction, hydrogen bond, or van der Waals force. By adding sodium chloride to the solution to shield the electrostatic effect, it was found that the adsorptive capacities of OVA and BSA were reduced to 86% and 76% of the initial values, confirming the role of electrostatic interaction in the adsorption of BSA and OVA to AlOOH nanorods (Supplementary Figure 8). It is worth noting that it has been suggested that OVA could be adsorbed on AlOOH nanorods through ligand exchange due to the existence of the two serine-bound phosphate groups^26,27^. However, according to our results, the adsorptive coefficient of OVA was similar to that of BSA. Therefore, the electrostatic effect could be dominant in the adsorption, with only a small amount of ligand exchanges during the adsorption process.

### Effect of surface hydroxyl on the mechanism of antigen adsorption

In order to better understand the correlation and adsorption mechanism, ITC was used to measure the thermodynamic parameters of the interaction between OVA and AlOOH nanorods. The interaction between OVA and AlOOH nanorods was exothermic, and was driven by enthalpy, although being unfavorable in terms of entropy (Fig. 4, Table 4)^28^. According to previous studies, the interaction between nanoparticles and proteins could involve two steps: solvent recombination, i.e., desolvation and solvation occur during the compounding process, and the formation of non-covalent or covalent bonds^29,30^. During the initial desolvation process, proteins and nanoparticles could approach each other, thus the hydration layer on the surface was destroyed, causing disordered discharge of water molecules and ions. The desolvation process was endothermic, thus led to an increase in entropy. When the nanoparticles and the antigens were coming closer, they interacted to produce a more stable complex through covalent or non-covalent interactions, and this process was enthalpy favorable. According to the thermodynamic parameters determined by ITC, the adsorption of OVA to AlOOH nanorods was directly driven by non-covalent interactions such as electrostatic interaction, hydrogen bonding or van der Waals forces, or covalent interactions such as ligand exchange. The negative contribution of entropy may be related to the loss of conformational entropy of the antigen, which may be due to the conformational limitation of amino acid residues after adsorption^31^. Surprisingly, for R3 or calcinated R1, the decrease of surface hydroxyl contents of the adjuvant resulted in the decrease of the adsorptive strength, while the heat release (ΔH) and entropy loss (ΔS) were increased. This phenomenon may be due to the difference in adsorptive strength that led to different degrees of desolvation^29,32^. It is reasonable to suggest that when nanorods with more surface hydroxyl groups adsorbed OVA, more water molecules and ions were released, which had a greater compensation for the loss of conformational entropy and the heat release during protein binding. In other words, although the negative enthalpy changes produced by the various interactions during the binding process drove the complexation of AlOOH nanorods and OVA, the process of desolvation directly affected the adsorptive strengths of antigens to nanorods with different hydroxyl contents. It is suggested that there were layers of water molecules on the surface of γ-AlOOH, and strong hydrogen bonds would be formed between water molecules^33^. The highly ordered structure with more hydroxyl groups on the surface of AlOOH increases the number of hydrogen bonds in the layer, thereby reducing the possibility of forming hydrogen bonds with adjacent water layers, making it easier for proteins to approach the surface of AlOOH nanorods. This speculation was also confirmed in Kang’s molecular simulation of protein adsorption on titanium dioxide with surface hydroxyl groups^34^.

**Fig. 6:**
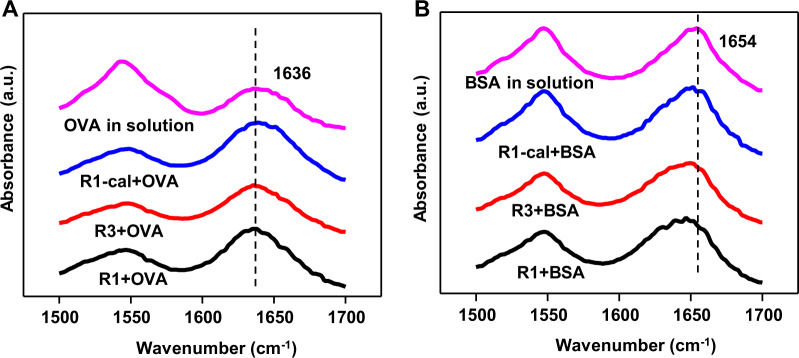
ATR-FTIR analysis of antigens. ATR-FTIR spectra of **A** OVA and **B** BSA in solution and adsorbed by AlOOH nanorods.

In addition to the effect of surface hydroxyl on the ligand exchange of HBsAg and the influence of water molecules mentioned above, the surface hydroxyl could also have an impact on other interactions. By examining the FTIR spectra of OVA in solution and adsorbed on AlOOH nanorods, it was found that the peak of the amide I region did not change significantly (Fig. 6A), indicating that the hydrogen bond between aluminum oxyhydroxide and OVA was not sufficient to drive adsorption. The surface hydroxyl groups on AlOOH nanorods may simply contribute to electrostatic interaction (Supplementary Figure 8) or van der Waals forces. Additionally, the surface hydroxyl groups may also promote the particle-protein interaction through ligand exchange^26,27^. However, the FTIR analysis of the peak of BSA in the amide I region after adsorption showed a significant shift to a lower wavenumber (Fig. 6B)^35,36^, and with the increase of surface hydroxyl content, the degree of shift also gradually increased. The transition to a lower wavenumber indicated an increase in hydrogen bonds, and the infrared frequency shift could be attributed to the formation of stronger hydrogen bonds between the surface hydroxyl groups of AlOOH nanorods and the amino acid residues of BSA.

### Characterization of structure and stability of adsorbed antigen

**Fig. 7:**
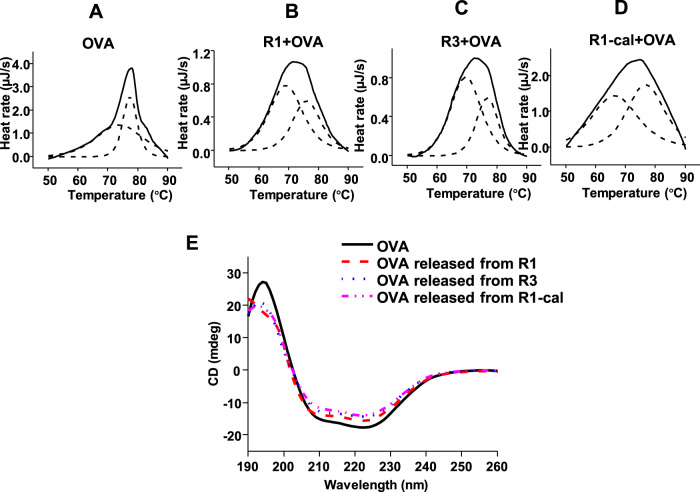
Analysis of the influence of AlOOH nanorods on the structure and stability of OVA. **A**–**D** Thermograms and peak fits (dashed line) obtained by DSC for OVA in solution and adsorbed by AlOOH nanorods. **E** CD spectra of native OVA and OVA released from AlOOH nanorods.

The surface hydroxyl display could affect the adsorption behavior of antigen. Thus, it is necessary to further evaluate if the change in surface hydroxyl contents could have an impact on the antigen stability in a vaccine. Differential scanning calorimetry (DSC) was used to study the structural thermal stability of the OVA adsorbed on engineered AlOOH nanorods. R1, R3, and calcinated R1 were selected to study the antigen stability. Compared with the antigen in the solution, the transition temperature (T_m_) and thermograms of the antigen adsorbed on the adjuvant were different (Fig. 7A–D, Table 6). The thermogram of OVA in solution was modeled by two component transitions centered at 74 °C and 77 °C, with 77 °C being the dominant one. When adsorbed on R1 and R3, the two T_m_ values of OVA were slightly reduced, and the peak at lower temperature showed a larger contribution, which indicated that the presence of AlOOH nanorods reduced the thermal stability of OVA. When adsorbed on R1, the T_m_ values of OVA were 69.07 °C and 76.01 °C, respectively. Compared with R1, the T_m_ values of OVA adsorbed on R3 was increased by about 0.5 °C, reaching 69.68 °C and 76.77 °C, which showed that adsorption on R3 with lower hydroxyl contents and specific surface area had better thermal stability. Similarly, the calcinated R1 was used to verify the role of hydroxyl groups. It was found that although the thermal stability of the lower peak (T_m_~66 °C) after adsorption was reduced, the 77 °C peak was still dominant, suggesting that fewer surface hydroxyl groups were beneficial to thermal stability of OVA on the adjuvant.

**Table 6:** The T_m_ values of the protein in the solution and adsorbed on the AlOOH nanorods.

Sample ID	T_m1_ (°C)	T_m2_ (°C)
OVA	74.04 ± 0.20	77.34 ± 0.04
AlOOH-R1 + OVA	69.07 ± 0.28	76.01 ± 0.19
AlOOH-R3 + OVA	69.68 ± 0.13	76.77 ± 0.08
AlOOH-R1-cal+OVA	66.54 ± 0.80	76.78 ± 0.32

In order to better understand the change of stability, circular dichroism (CD) was used to determine the secondary structure of OVA released after being adsorbed on the AlOOH nanorods. The CD spectrum showed that OVA bound weakly in its native conformation^37^. The secondary structure of OVA was composed of 21% α-helix, 17% β-sheet, 15% β-turn, and 46% disordered conformations. Structural analysis showed that after OVA was released from R1, the α-helix and β-turn contents decreased slightly, while the β-sheet increased from 17% to 37%, and other undefined disordered structures decreased from 46% to 34%. The β-sheet contents of OVA released from R3 only increased by 4%. Similarly, when OVA was released from calcinated R1 with fewer surface hydroxyl groups and little changes in specific surface area, only limited changes have taken place in the β-sheet structures. The AlOOH nanorods with more surface hydroxyl groups could lead to greater changes in the secondary structure, which also explains the lower thermal stability of antigens adsorbed on the nanorods with more surface hydroxyl groups (Fig. 7E, Supplementary Table 10). The strong interaction between nanorods and OVA may cause the destabilization of protein structure, and the released protein was prone to aggregation resulting in an increase in β-sheets and a decrease in disordered structure to stabilize the conformation^37–40^. The results here clearly showed that the surface hydroxyl groups could enhance the adsorptive strength of the antigen on the adjuvant, but at the same time, it is more likely to cause destabilization of the absorbed antigen. Whether the changes in adsorptive strength and stability have a positive impact on the immunogenicity of the vaccine depends on the immune mechanism of the antigen-adjuvant complexes^4,6,8,41^, which requires further detailed studies.

## Conclusion

In summary, an engineered approach was developed to evaluate the antigen adsorption behavior on aluminum-based adjuvants in vaccine formulations. By controlling the characteristics of AlOOH nanorods, the antigen adsorption was studied and utilizing a diversity of physicochemical and biophysical characterizations. It is demonstrated that the specific surface area of AlOOH nanorod is positively correlated with the adsorptive capacity, and the surface hydroxyl is not only positively correlated with the strength of adsorption, but also causes structural changes and even partial instability of the antigen after adsorption. The overall goal of our findings is to improve the design of vaccine adjuvants, and promote the development of optimal vaccine formulations.

## Methods

### Materials and reagents

The Alhydrogel^®^ was obtained from InvivoGen (San Diego, California). The bovine serum albumin (BSA) and Ovalbumin (OVA) were purchased from Sigma (St Louis, MO). The Hepatitis B surface antigen (HBsAg) was purchased from North China Pharmaceutical Group Genetech Biotechnology Co., Ltd. (Shijiazhuang, China). The SARS-CoV-2 Spike Protein’s receptor-binding domain (RBD) was purchased from Genscript (Nanjing, China). The potassium hydroxide, sodium hydroxide, MOPS [3-(N-morpholino) propane sulfonic acid], MOPS sodium salt were obtained from Sangon (Shanghai, China). The hydrochloric acid was received from Kemiou (Tianjin, China). The Potassium nitrate was obtained from Damao Chemical Reagent Factory (Tianjin, China). The Pierce BCA protein assay kit was purchased from Thermo Scientific (Logan, UT).

### Synthesis of aluminum hydroxide (γ-AlOOH) nanorods

The synthesis of AlOOH nanorods was conducted using hydrothermal method^12,13^. In a typical reaction, 1.3933 g of aluminum (III) nitrate nonahydrate [Al(NO_3_)_3_·9H_2_O] was added in 20 mL of pure water. Then, 0.238 mL of ethylenediamine (EDA) was added to the solution while stirring. After stirring for 15 min, the reaction mixture was transferred to a Teflon-lined stainless steel auto-clave, the temperature was maintained at 160 to 200 °C in an electric oven for 16 h. The final product was dried at 60 °C overnight before use. The AlOOH synthesized at 160 °C was placed in a muffle furnace at 350 °C for one hour to remove surface hydroxyl groups. To prevent the aggregation of the calcined sample from affecting antigen adsorption, the sample was allowed to precipitate to remove the larger aggregated samples before taking for subsequent experiments.

### Physicochemical characterization of AlOOH nanorods

A transmission electron microscope (JEM-2100, JEOL, Japan) was used to determine the morphologies and primary sizes of AlOOH nanorods. A X-ray powder diffraction (XRD, Rigaku D/Max 2400 type X-ray spectrometer) equipped with CuKα radiation (λ = 1.54178 Å) was used to determine the phase and crystallinity of AlOOH nanorods. A ZetaPALS instrument (90Plus Zeta, Brookhaven, USA) was used to measure the hydrodynamic sizes and zeta potentials of AlOOH nanorods. A surface area and pore size analyzer (AUTO SORB-1-MP, QUANTOCHROME, USA) was used to measure the specific surface area of the AlOOH nanorods. The isoelectric point of the AlOOH nanorods was measured using KNO_3_ as the buffer solution, and its pH was adjusted by proper amounts of potassium KOH (0.01-1 M) and HCl (0.01–1 M) under the condition that ionic strength remained constant. The zeta potentials of AlOOH nanorods in KNO_3_ buffer at different pH was determined. The hydroxyl contents on the surface of AlOOH nanorods is weakly acidic. Potentiometric titration was used to measure the hydroxyl content on AlOOH nanorods^42^, the mixture of 200 mg of AlOOH nanorods with 35 mL of pure water were titrated with 0.05 M KOH. 35 mL of pure water was used as blank control. The equivalence points (EP) were determined using the maxima of the first derivate of the titration curve (dpH/dVolume_KOH_). The amount of surface hydroxyl contents, n(Al-OH), was calculated by using the following formula, n(Al-OH) = nKOH[EP] -nH_2_O[EP].

### Determination of adsorption of antigens on AlOOH nanorods

10 mM of MOPS and 50 mM of NaCl buffer were prepared, and the pH of the buffer was adjusted to 7.4 with 0.1 M of NaOH. For BSA and OVA, 0.5 mL of proteins working solution (1–6 mg/mL) and 0.5 mL of nanorods suspension in desired buffer (1.7 mg Al/mL) were mixed in a microcentrifuge tube. For HBsAg, 0.5 mL of HBsAg working solution (50–300 μg/mL) and 0.5 mL of AlOOH nanorod suspension in prepared buffer (100 µg Al/mL) was mixed in a microcentrifuge tube. For RBD, due to logistical reasons, the volume of the AlOOH nanorods and antigen was reduced by half, and the rest of the experimental steps were the same as those for HBsAg. The adsorption was performed by mixing of the antigen and adjuvant (as described above) at room temperature for 30 min. The samples were then centrifuged at 7600 × *g* for 25 min, and the unadsorbed antigens free in solution was determined by BCA assay. The Langmuir equation was used to describe adsorption of antigen on the nanorods, in which the solute (antigen) adsorbed on the nanorods forms a monolayer. The Langmuir’s isotherm equations were fitted for the adsorption of BSA, OVA, HBsAg, and RBD^5^. By constructing adsorption isotherms, the amount of the proteins that can be adsorbed onto the nanorods was determined. The adsorptive capacity was taken from the plateau of the experimental adsorption isotherm. The data analysis of the adsorption isotherms by Langmuir fitting was used to determine the monolayer adsorptive capacity and the adsorptive coefficient of protein to particle surface. The monolayer adsorptive capacity represented the mass of antigen adsorbed as a monolayer per mass of aluminum and the adsorptive coefficient represented the strength of the adsorption force. Whether the data from the adsorption isotherm conforming to the Langmuir equation was determined by the linear fitting degree, R^2^.

### Biothermodynamic analysis of antigen adsorption to AlOOH nanorods

Isothermal Titration Calorimetry (ITC) measurements were performed on a Nano ITC calorimeter (TA, USA). Both OVA antigen and AlOOH nanorods were diluted in 10 mM of MOPS buffer (pH 7.4). 25 injections were titrated with 15 mg/mL of OVA to a sample cell containing 1.7 mgAl/mL of AlOOH nanorods in each experiment. Each injection of 2 µL was performed with an interval of 400 s. All titrations were performed with a stirring speed of 300 rpm at 25 °C. The background of the protein to buffer titration was subtracted from the raw data to exclude the influence of the heat of dilution. The ITC data were analyzed by Lanuch Nanoanalyze software (TA, USA), and the adsorptive strength and thermodynamic parameters of each interaction were calculated. The data were fitted to a standard model.

### ATR-FTIR spectroscopy of antigen adsorbed on AlOOH nanorods

Adsorption of antigen on AlOOH nanorods was determined by Fourier transform infrared (FTIR) spectroscopy (Spectrum 3, PerkinElmer, UK) with a 45° ZnSe attenuated total reflectance (ATR) crystal^43^. Briefly, 0.4 mL of AlOOH nanorods suspension (1 mg/mL) was dispersed on the ZnSe sample cell to form a uniform film on the crystal surface. After equilibrating the AlOOH nanorods coating with the background MOPS buffer solution for 1 h, a spectrum was collected as the blank. Then, the buffer solution on the crystal surface was discarded, and 0.7 mL of antigen solution (0.01 mg/mL) was placed to the ZnSe sample cell to adsorb on the formed AlOOH film. The antigen infrared spectrum was collected until there is no significant change. As a control, the concentration of OVA in the solution was 5 mg/mL, and it was directly placed on the ZnSe sample cell to obtain the spectrum. Each spectrum was scanned for 32 times at a 4 cm^−1^ resolution over the range of 4000–700 cm^−1^.

### Determination of thermal transition temperature (T_m_) by differential scanning calorimetry (DSC)

The T_m_ of the OVA and BSA antigen in solution and adsorbed onto the nanorods were monitored by Nano DSC (TA, USA). Antigen completely adsorbed nanorods were prepared. Samples of OVA-AlOOH nanorods with 0.5 mg/mL of OVA and 2 mg Al/mL of AlOOH nanorods in 10 mM of MOPS buffer were analyzed. For the analysis of BSA-AlOOH nanorods, the concentration of BSA and AlOOH nanorods were increased to 1 mg/mL and 4 mg Al/mL, respectively. The samples were scanned from 10 °C to 100 °C at a rate of 1 °C /min. Before data analysis, the thermogram of the buffer was used as a blank to subtract from the separate antigen solution. Similarly, the AlOOH nanorods of the same concentration were diluted into the buffer to subtract from the adsorbed antigens. DSC data were analyzed using Lanuch Nanoanalyze software (TA, USA). For OVA, whether in solution or in the OVA-AlOOH nanorods complexes, transition temperatures were determined by using a two-state model. BSA thermogram in solution was fitted using a single peak two-state model. A three-peak model was used to provide fit for BSA adsorbed to AlOOH nanorods.

### Secondary structure characterization of antigen

Circular Dichroism (CD) spectra of natural proteins and adsorbed proteins were measured in the wavelength range of 190–260 nm using a multifunctional circular dichroic spectrometer (MOS-500, BioLogic Science Instruments, France). Similarly, samples of antigens completely adsorbed on AlOOH nanorods were prepared as described above. The concentration of OVA was 1.6 mg/mL, and the concentration of AlOOH nanorods was 4.8 mgAl/mL. Adsorbed proteins were released by adding 0.2 M PBS to a final protein concentration of 400 µg/mL. After the sample was incubated for 30 min, the supernatant was taken for CD detection. Free OVA samples were prepared at the same concentration in 0.2 M PBS. The sample was scanned three times in a quartz cell with a path length of 0.1 cm and averaged spectrum was used for further data analysis. Before the sample measurement, the CD spectrum of the control sample containing all components except protein was subtracted from each spectrum^44^. The BeStSel server was used to analyze the contents of different secondary structures of the released antigen and the original antigen in the solution.

### Statistical analysis

Triplicate samples were included for all experiments. All the experiments were performed for two to three times. The values represent average ± SD. Statistical significance was determined by two-tailed Student’s *t*-test for two-group analysis.

### Reporting summary

Further information on research design is available in the Nature Research Reporting Summary linked to this article.

## Supplementary information

Supporting Information

REPORTING SUMMARY

## Data Availability

All datasets generated during and/or analyzed during the current study are available from the corresponding author on reasonable request.
