# Supplementary material for: Engineering the hydroxyl content on aluminum oxyhydroxide nanorod for elucidating the antigen adsorption behavior
Source: NPJ Vaccines. 2022 Jun 23;7:62. doi: 10.1038/s41541-022-00495-9 (PMC9226065; doi:10.1038/s41541-022-00495-9)
Supplement: Supplementary file 1 — Supporting Information [file 41541_2022_495_MOESM1_ESM.pdf]

Supplementary information

Engineering the Hydroxyl Content on Aluminum Oxyhydroxide Nanorod for Elucidating the Antigen Adsorption Behavior

Ge Yu[1,2], Zhihui Liang[1,2], Zilan Yu[2], Min Li[1,2], Wenqi Yang [1,2], Yawei Zhang[2], Yuhang Zhao[2], Cheng Yang[3], Changying Xue[4], Li Shi[5], Bingbing Sun[1,2]*

Address and corresponding author

[1]State Key Laboratory of Fine Chemicals, Dalian University of Technology, 2 Linggong Road, 116024, Dalian, China.

[2]School of Chemical Engineering, Dalian University of Technology, 2 Linggong Road, 116024, Dalian, China.

[3]School of Chemistry, Dalian University of Technology, 2 Linggong Road, 116024, Dalian, China.

[4]School of Bioengineering, Dalian University of Technology, 116024, Dalian, China

[5]Immune-Path Biotechnology (Su Zhou) Co., Ltd., Building A, 8 Chang Ting Road, DaXin Industry Park, 215151, Su Zhou, Jiang Su, China.

[*]Address correspondence:

Bingbing Sun bingbingsun@dlut.edu.cn

Figures

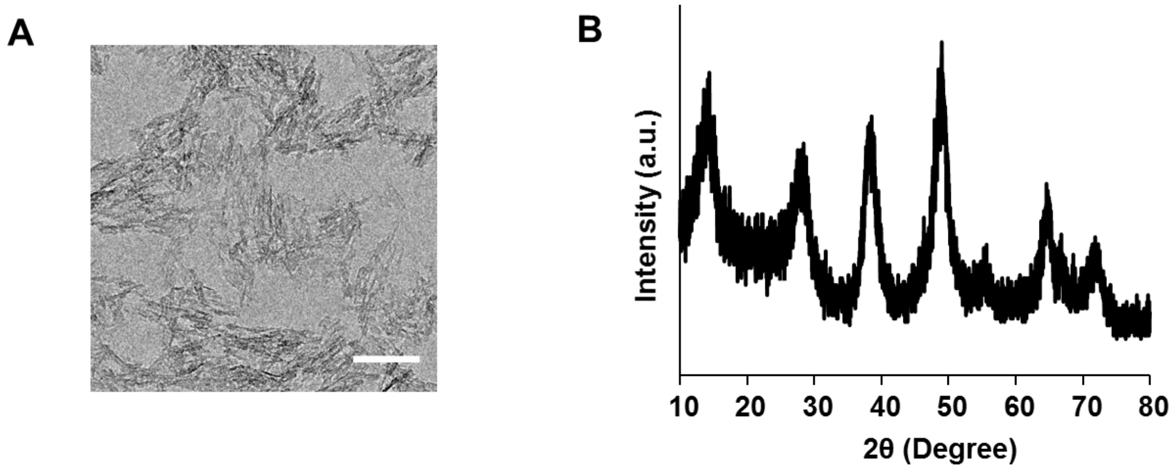

Supplementary Figure 1. Characterization of physicochemical properties of Alhydrogel®. (A) TEM analysis and (B) XRD patterns. The scale bar is 100 nm.

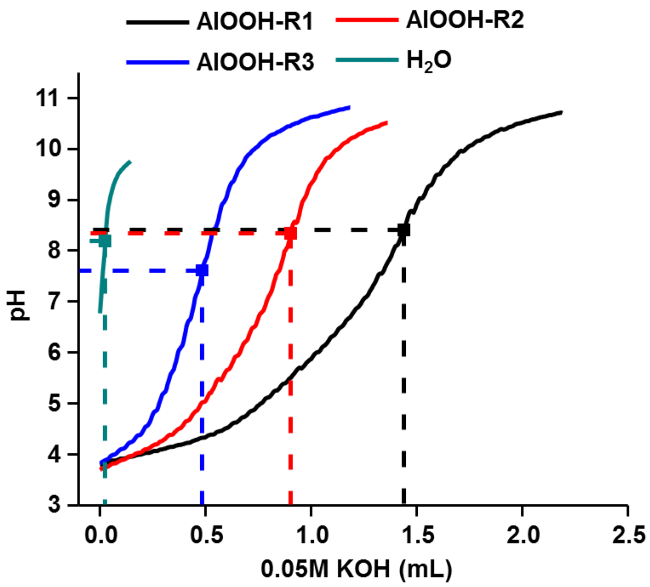

Supplementary Figure 2. Titration curves of AlOOH-R1, AlOOH-R2, AlOOH-R3 in water and the reference (water without particles). The dash lines show the pH and KOH consumption at the equivalence points.

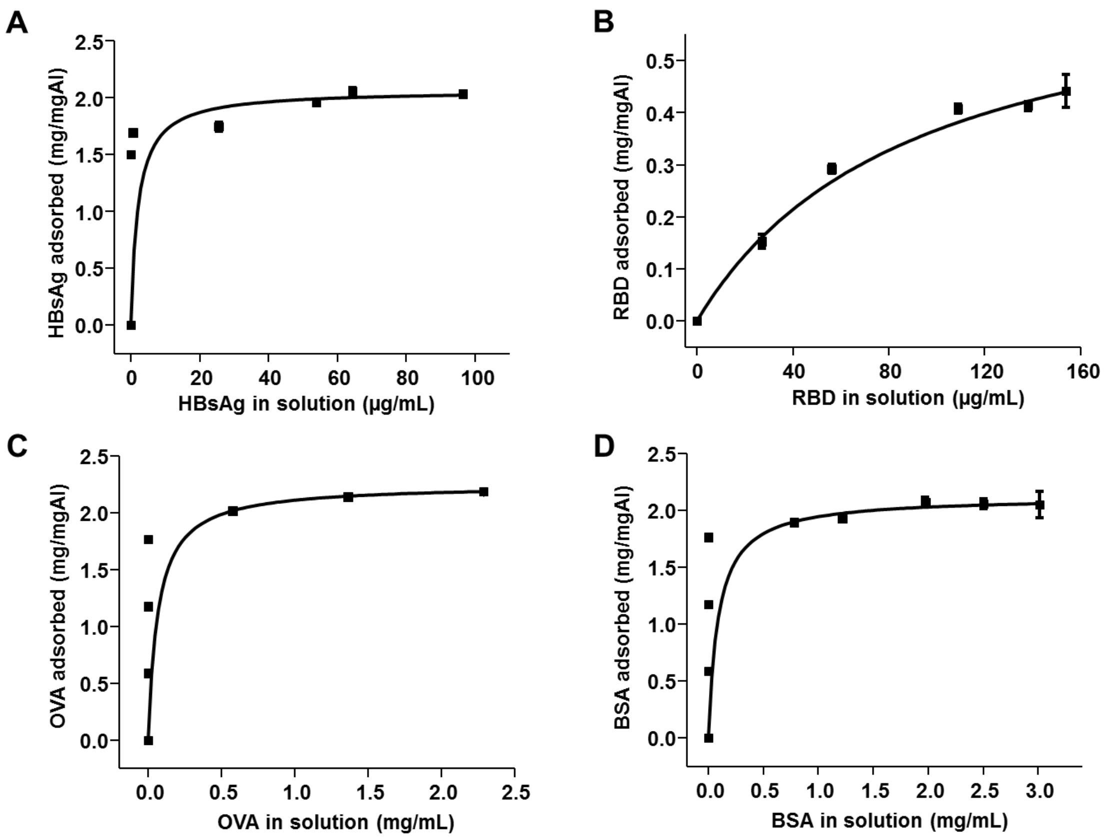

Supplementary Figure 3. Adsorption isotherms of (A) HBsAg, (B) RBD, (C) OVA, (D) BSA by Alhydrogel® at pH 7.4.

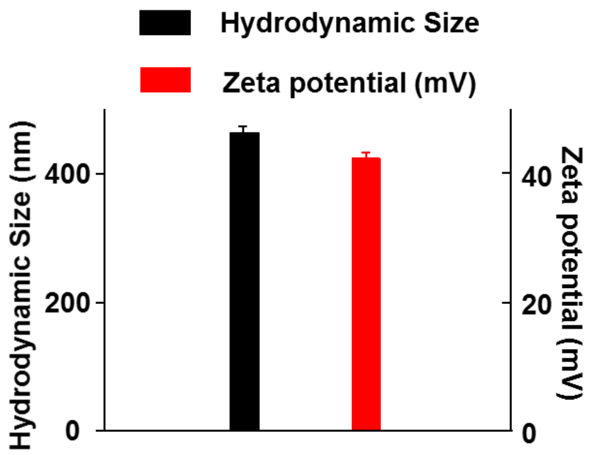

Supplementary Figure 4. Hydrodynamic size and zeta potential of the AlOOH-R1 after Calcination. AlOOH-R1 was obtained by treating AlOOH nanorods at 350 ℃ for 1 hour.

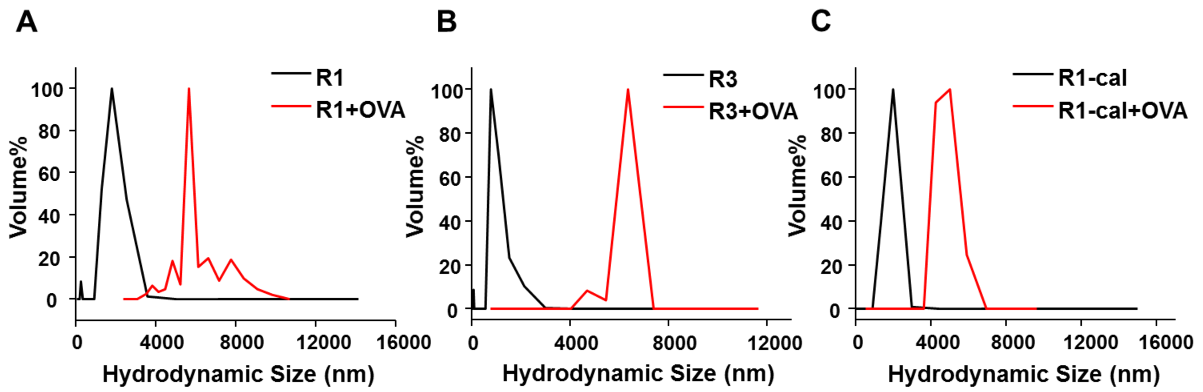

Supplementary Figure 5. The hydrodynamic particle size distribution of (A) R1, (B) R3, and (C) R1-cal alone and supplemented with OVA, respectively.

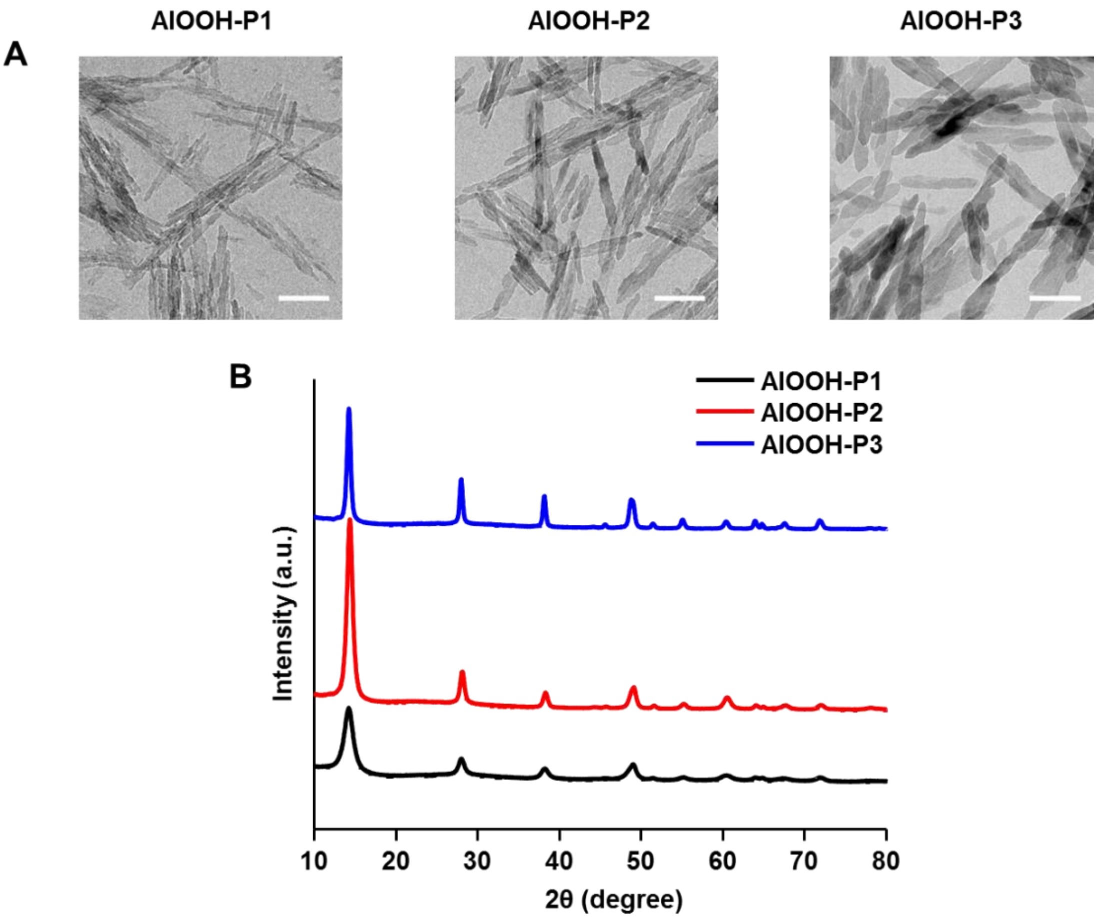

Supplementary Figure 6. (A) TEM analysis and (B) XRD patterns of in house-synthesized AlOOH nanorods. TEM images of AlOOH nanorods at a synthesis time of 2 h (P1), 6 h (P2), and 24 h (P3). The scale bar is 100 nm.

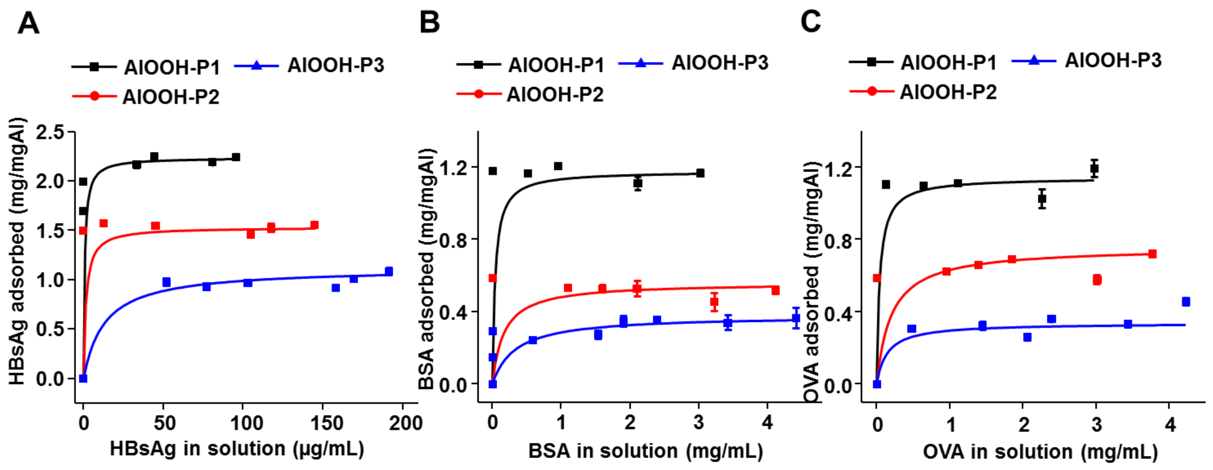

Supplementary Figure 7. Adsorption isotherms of (A) HBsAg, (B) BSA, and (C) OVA by AlOOH nanorods synthesized with different synthesis times at 200 ℃.

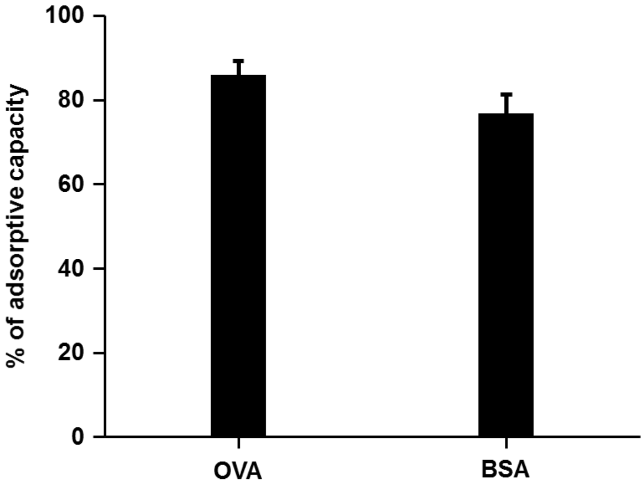

Supplementary Figure 8. Effect of ionic strength on the adsorptive capacity of BSA and OVA at a pH value of 7.4. 1.7 mgAl/mL of AlOOH-R1 was used to adsorb 6 mg/mL of model antigen in MOPS buffer with 0.05 M of sodium chloride and 0.75 M of sodium chloride, respectively. The ratio of adsorptive capacity in 0.75 M of sodium chloride to that in 0.05 M of sodium chloride was expressed as % of adsorptive capacity.

Tables

Supplementary Table 1. The particle size of AlOOH nanorods measured by TEM.

Sample ID	Particle size (nm)
AlOOH-R1	222±40
AlOOH-R2	216±33
AlOOH-R3	218±33

Supplementary Table 2. Hydrodynamic sizes, zeta potentials, point of zero charges (PZC), specific surface areas , surface hydroxyl contents and the width at half height (WHH) of the (020) of Alhydrogel®.

Sample ID	Hydrodynamic size in water (nm)	Zeta potential in water (mV)	PZC	Specific surface area(m^2/g)	Hydroxyl content (mmol/g)	WHH (2θ)
Alhydrogel®	503±9	27±2	9.61 ±0.04	270.8 ±11.7	NA	1.74±0.14

Supplementary Table 3. The zeta potential of AlOOH nanorods at neutral pH in 10mM of KNO_3 buffer.

Sample ID	Zeta potential (mV)
AlOOH-R1	37±1
AlOOH-R2	32±2
AlOOH-R3	25±3
AlOOH-R1-cal	26±2

Supplementary Table 4. Statistical differences in adsorption parameters of AlOOH nanorods for HBsAg, RBD, OVA and BSA. $^{*}p < 0.05$, $^{**}p < 0.01$ and $^{***}p < 0.001$ compared to AlOOH-R1.

Antigen	Adjuvant	Adsorptive capacity (mg/mgAl)	Adsorptive coefficient (mL/mg)	Monolayer adsorptive capacity (mg/mgAl)
HBsAg	AlOOH-R1	-	-	-
	AlOOH-R2	*	*	*
	AlOOH-R3	***	*	***
BSA	AlOOH-R1	-	-	-
	AlOOH-R2	**	-	**
	AlOOH-R3	**	***	**
OVA	AlOOH-R1	-	-	-
	AlOOH-R2	*	-	**
	AlOOH-R3	**	*	***
RBD	AlOOH-R1	-	-	-
	AlOOH-R2	*	*	**
	AlOOH-R3	***	**	***

Supplementary Table 5. Adsorption parameters for HBsAg, RBD, OVA, and BSA by Alhydrogel® at pH 7.4.

Adjuvant	Antigen	Adsorptive capacity (mg/mgAl)	Coefficient of determination (R^2)	Adsorptive coefficient (mL/mg)	Monolayer adsorptive capacity (mg/mgAl)
Alhydrogel®	HBsAg	2.03 ±0.03	0.99 ±0.00	475 ±53	2.07 ±0.08
	BSA	2.05 ±0.07	0.99 ±0.00	11 ±4	2.12 ±0.02
	OVA	2.18 ±0.08	0.99 ±0.00	15 ±2	2.25 ±0.06
	RBD	0.44 ±0.01	0.97 ±0.01	11 ±1	0.70 ±0.01

Supplementary Table 6. Adsorption parameters for OVA and BSA by AlOOH-R1 and AlOOH-R1-cal nanorods at different concentrations.

Antigen	Concentration	Adjuvant	Adsorptive capacity (mg/mgAl)	Adsorptive coefficient (mL/mg)	Monolayer adsorptive capacity (mg/mgAl)
OVA	High	AlOOH-R1	1.04±0.06	21±5	1.05±0.06
		AlOOH-R1-cal	0.94±0.02	7±4	0.90±0.05
	Low	AlOOH-R1	1.41±0.07	219±28	1.42±0.05
		AlOOH-R1-cal	1.26±0.01	82±8	1.28±0.03
BSA	High	AlOOH-R1	1.12±0.07	37±2	1.10±0.07
		AlOOH-R1-cal	0.77±0.03	5±2	0.82±0.01
	Low	AlOOH-R1	1.30±0.08	195±13	1.27±0.11
		AlOOH-R1-cal	1.13±0.11	70±4	1.18±0.11

Supplementary Table 7. Statistical differences in hydroxyl contents, specific surface area and adsorption parameters of AlOOH R1 and AlOOH R1-cal for HBsAg, OVA and BSA. *p < 0.05, **p < 0.01 and ***p < 0.001 compared to AlOOH-R1.

Antigen	Adjuvant	Hydroxyl content (mmol/g)	Specific surface area (m^2/g)	Adsorptive capacity (mg/mgAl)	Adsorptive coefficient (mL/mg)
HBsAg	AlOOH-R1	-	-	-	-
	AlOOH-R1-cal	***	-	-	*
BSA	AlOOH-R1	-	-	-	-
	AlOOH-R1-cal	***	-	**	***
OVA	AlOOH-R1	-	-	-	-
	AlOOH-R1-cal	***	-	-	*

Supplementary Table 8. Hydrodynamic size, zeta potential, point of zero charge (PZC), specific surface area and surface hydroxyl contents of AlOOH nanorods prepared with different synthesis times at 200 ℃.

Sample ID	Hydrodynamic size in water (nm)	Zeta potential in water (mV)	PZC	Hydroxyl content (mmol/g)	Specific surface area (m^2/g)	WHH (2θ)
AlOOH-P1	195±6	49±1	10.27±0.01	0.44±0.02	174.6±21.0	1.25±0.04
AlOOH-P2	197±3	41±0	9.95±0.00	0.22±0.02	118.0±7.2	0.83±0.05
AlOOH-P3	178±5	40±2	9.62±0.01	0.13±0.01	71.8±4.6	0.51±0.03

Supplementary Table 9. Adsorption parameters for HBsAg, BSA, and OVA by AlOOH nanorods prepared with different synthesis times at 200 ℃.

Antigen	Adjuvant	Adsorptive capacity (mg/mgAl)	Coefficient of determination (R^2)	Adsorptive coefficient (mL/mg)	Monolayer adsorptive capacity (mg/mgAl)
HBsAg	AlOOH-P1	2.24±0.01	0.99±0.00	1391±430	2.24±0.01
	AlOOH-P2	1.55±0.04	0.99±0.00	657±204	1.54±0.11
	AlOOH-P3	1.08±0.07	0.99±0.00	77±35	1.12±0.07
BSA	AlOOH-P1	1.16±0.03	0.99±0.00	25±10	1.18±0.01
	AlOOH-P2	0.58±0.01	0.96±0.01	6±1	0.56±0.01
	AlOOH-P3	0.37±0.04	0.98±0.01	3±1	0.38±0.02
OVA	AlOOH-P1	1.19±0.03	0.99±0.00	24±6	1.14±0.02
	AlOOH-P2	0.72±0.03	0.99±0.01	5±2	0.76±0.05
	AlOOH-P3	0.35±0.04	0.94±0.04	7±1	0.34±0.04

Supplementary Table 10. Percentage of secondary structures of OVA in solution and adsorbed on the AlOOH nanorods.

Sample ID	Helix %	Beta %	Turn %	Others %
OVA	21.2±2.6	17.4±0.2	15.4±1.0	46.2±3.8
OVA released from R1	17.8±2.8	37.2±1.2	10.8±0.5	34.2±2.2
OVA released from R3	20.5±3.7	23.0±3.6	14.2±2.1	42.5±2.0
OVA released from R1-cal	20.4±1.7	26.2±0.9	12.7±0.4	40.8±2.2
